# Augmented reality in medical education: students’ experiences and learning outcomes

**DOI:** 10.1080/10872981.2021.1953953

**Published:** 2021-07-14

**Authors:** Poshmaal Dhar, Tetyana Rocks, Rasika M Samarasinghe, Garth Stephenson, Craig Smith

**Affiliations:** a Institute for Innovation in Mental and Physical Health and Clinical Translation, School of Medicine, Faculty of Health, Deakin University, Geelong, Australia; b Institute for Innovation in Mental and Physical Health and Clinical Translation, Food and Mood Centre, School of Medicine, Faculty of Health, Deakin University, Geelong, Australia

**Keywords:** Augmented reality, medical education, education technology, online learning, learning outcomes

## Abstract

Augmented reality (AR) is a relatively new technology that allows for digitally generated three-dimensional representations to be integrated with real environmental stimuli. AR can make use of smart phones, tablets, or other devices to achieve a highly stimulating learning environment and hands-on immersive experience. The use of AR in industry is becoming widespread with applications being developed for use not just for entertainment and gaming but also healthcare, retail and marketing, education, military, travel and tourism, automotive industry, manufacturing, architecture, and engineering. Due to the distinct learning advantages that AR offers, such as remote learning and interactive simulations, AR-based teaching programs are also increasingly being adopted within medical schools across the world. These advantages are further highlighted by the current COVID-19 pandemic, which has caused an even greater shift towards online learning. In this review, we investigate the use of AR in medical training/education and its effect on students’ experiences and learning outcomes. This includes the main goals of AR-based learning, such as to simplify the delivery and enhance the comprehension of complex information. We also describe how AR can enhance the experiences of medical students, by improving knowledge and understanding, practical skills and social skills. These concepts are discussed within the context of specific AR medical training programs, such as HoloHuman, OculAR SIM, and HoloPatient. Finally, we discuss the challenges of AR in learning and teaching and propose future directions for the use of this technology in medical education.

## Introduction

Augmented reality (AR), a type of mixed reality, is a real-world based experience that is enhanced by digital objects or information. Barsom, Graafland & Schijven (2016) describe augmented reality as “ … an interactive virtual layer on top of reality’. Practically, this is usually achieved via a head-set or tablet-style devise (including smart phones) in which the digital object is created and is surrounded by the real environment. In addition to visual digital stimuli, enhancement of reality in education can be also achieved by introducing auditory, haptic (touch), and even olfactory information or feedback [1]. By presenting only partial augmentations of the real environment, this mixed reality allows to precisely control the level of exposure carefully shaping the learning experience. This is different from virtual reality (VR), in which the entire environment is digitally created. However, both AR and VR sit at different ends of the mixed reality continuum, which was acknowledged over 25 years ago when this field was first emerging [2].

AR is a rapidly developing technology. Due to its flexibility in integrating physical and virtual environments, AR-based programs are increasingly used in education, including medical education and training. The use of this technology provides various means of delivering learning content and enhancing students’ experiences.

### Brief history of augmented reality use in medical education

Due to the advantages that AR technology offers, several programs have been successfully implemented in the field of medicine. Broadly, these can be categorised into two subgroups. The first involves *treatment* programs which help patients and/or practitioners within a hospital or clinical setting, such as therapies, rehabilitation, or surgical procedures. The second includes *training* programs which are instead designed to aid teaching and learning outcomes within the academic university setting [1]. This review will focus on the latter of these two categories, and will explore how they have taken advantage of key features of this technology to develop or improve knowledge, learning, and skill outcomes.

Before the use of computers in medical education, text-books, lectures, cadavers, anatomical models and live patients were some of the only pedagogical tools available. Basic computer-assisted anatomy programs started to appear in the early 1990s [3,4], and were often accompanied with multimedia approaches such as the ‘Slice Of Life’ videodiscs that served as a visual anatomy encyclopaedia [5]. Continuing advancements in hardware allowed for presentation software such as Microsoft PowerPoint to mostly replace blackboards and overhead projections in the 1990s [6], while the World Wide Web made the Visible Human Project [7] and similar programs like the Visible Embryo Project [8] possible. The mid-1990s also saw the use of computer-based stereoscopy, in which slightly offset two dimensional images are displayed in each eye to give the illusion of three dimensional depth [9]. Although the first head-mounted display was developed in the late 1960s [10], the adoption of VR within medical education has required more recent technological advancements such as the availability of modern head-mounted displays including Google Glass, Microsoft HoloLens, Oculus Rift VR, and the Samsung Gear VR [11]. AR has also benefited from recent advancements in handheld smartphone and tablet devises, which not only improve the power of such programs, but also expand their accessibility out of traditional learning spaces and into the hands of the learner [12]. This feature of AR has become particularly important during the current COVID-19 pandemic, which caused restricted face-to-face access to many university and other learning spaces [13].

## Materials and methods

In this narrative review, we conducted an extensive literature review using searches within the following databases: PubMed, Google Scholar, and Scopus. Keywords including ‘Augmented reality’, ‘Medical education’, ‘Students experiences’, and ‘Learning outcomes’ were used to search for articles published in English, and those from the last 10 years were favoured. As this was not a systematic review, author discretion was used in selecting appropriateness, and a wider scope of topics is addressed (for an example of a systematic review that addresses a more narrow subject topic within this area, see [14]). We also conducted a complimentary search of the reference lists of key articles.

## Results

### Enhancement of student experiences and learning outcomes with AR-based medical programs

**Figure 1.**
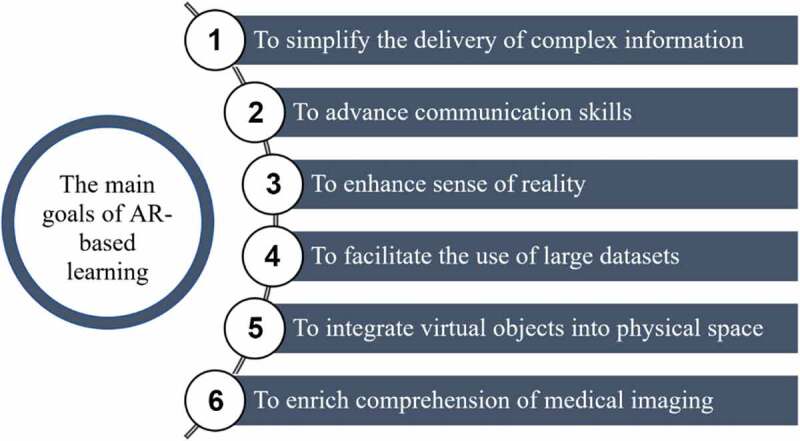
The main goals of augmented reality in medical education [16]

AR-based training provides a vast potential to effectively and efficiently prepare medical professionals for the real world of practice [15]. Along with offering a safe educational environment and addressing specific professional skills, AR programs for learning in medicine are employed to enhance learners’ experiences, as described by Salehahmadi and Hajialiasgari [16] (Figure 1).

AR learning is commonly associated with highly positive subjective personal experiences, and can be fun and interesting to use. It is for similar reasons that AR games such as Pokemon Go have been so successful [17]. Moreover, AR can enhance learning delivery, presentation and the utilisation of sensory systems, which are three crucial elements of Mayer’s cognitive theory of multimedia learning [18]. Due to the high level of digital literacy common amongst University students and their familiarity with using tablets and smartphones, students often report that they feel confident with adopting AR alternatives to traditional learning approaches, such as flashcards [19]. The ability to use devices that students already own also facilitates self-paced learning, and non-headset VR programs are less likely to cause adverse effects compared to VR while still achieving similar learning benefits. For example, Moro and colleagues showed that an AR structural anatomy program ran on a tablet achieved similar learning outcomes (e.g., anatomical knowledge test results) compared to a headset VR equivalent. Importantly however, the AR tablet version was less prone to adverse effects including general discomfort, headache, dizziness, nausea and disorientation, and was also less likely to cause eye-related problems such as blurred vision [20].

**Figure 2.**
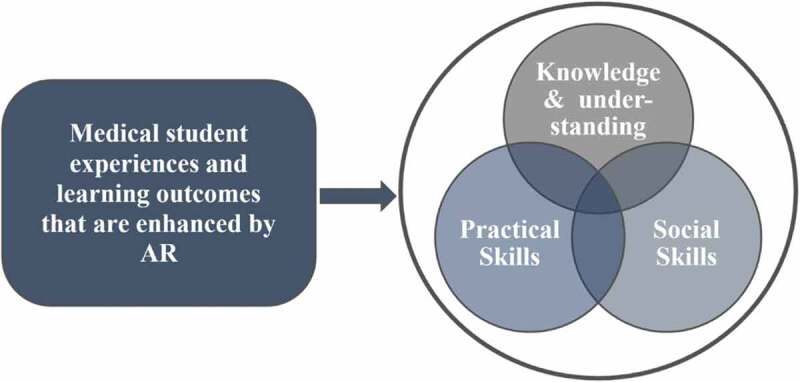
Three main domains of medical student experiences and learning outcomes that are enhanced by AR-based programs

Due to the ability of AR-based learning to support students’ experiences, not surprisingly, this can also translate into improved learning outcomes [1]. Students who successfully complete learning activities enhanced by AR programs are more likely to achieve both enhanced theoretical knowledge and practical skills. AR-based learning boosts outcomes in several main aspects of training, including professional knowledge, cognitive and practical skills, social skills, innovation, competence, and creativity [21]. Here, we focus on the effect of AR-based programs on students’ experience and learning outcomes in relation to the following three domains of impact: knowledge and understanding, practical skills, and social skills (Figure 2).

### AR enhancement of knowledge and understanding

**Figure 3.**
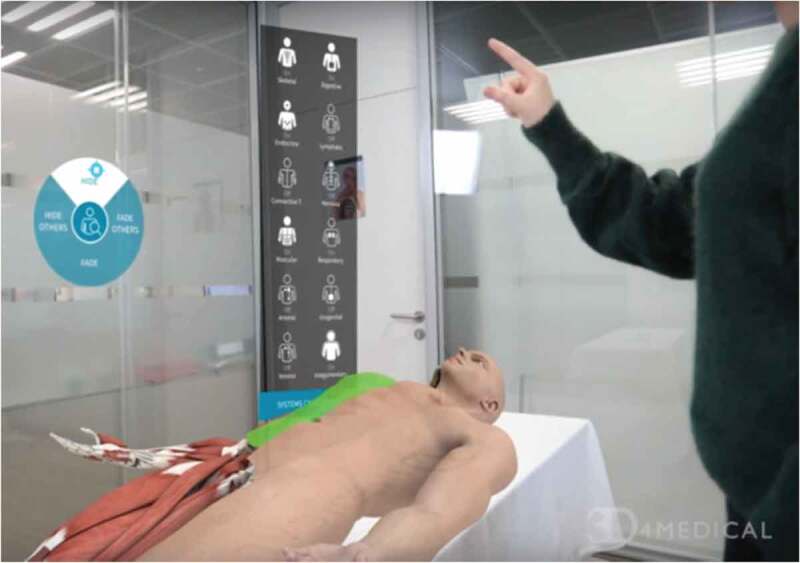
The AR app ‘HoloHuman’ showing a virtual cadaver placed on a real examination table. The moderator (shown) is able to interact with the model and user interface through the use of a HoloLens headset. Structures, organs and systems can be examines individually or in combination and are fully supported by visual narrative and digital dissection tools (image courtesy of 3D4 Medical from Elsevier, 2020; https://3d4medical.com/apps/holohuman)

Medical education is associated with an enormous amount of information pertaining to human anatomy and bodily function [22]. Learning this information has been greatly aided with the development of a plethora of digital programs, for example ‘virtual cadavers’ (Figure 3). Rather than being accessed via a traditional computer mouse, keyboard and screen, AR is able to enhance the way in which medical students interact with digital anatomical representation at all angles, providing a more immersive experience that ultimately aids knowledge and understanding [23].

An important advantage of such programs is that they allow easy manipulation of the digital subject, so that spatial inter-relationships can be identified and explored in three-dimensional space. In regards to anatomy, this for example allows for complicated branching nerve and blood vessel paths to be examined in isolation, which is difficult to do with traditional cadavers as these structures lose shape if dissected away from the surrounding tissue that supports them. Learning the names for the multitude of anatomical structures is also aided by the ability to select regions/structures of interest, and access a range of other information pertaining to them [24].

**Figure 4.**
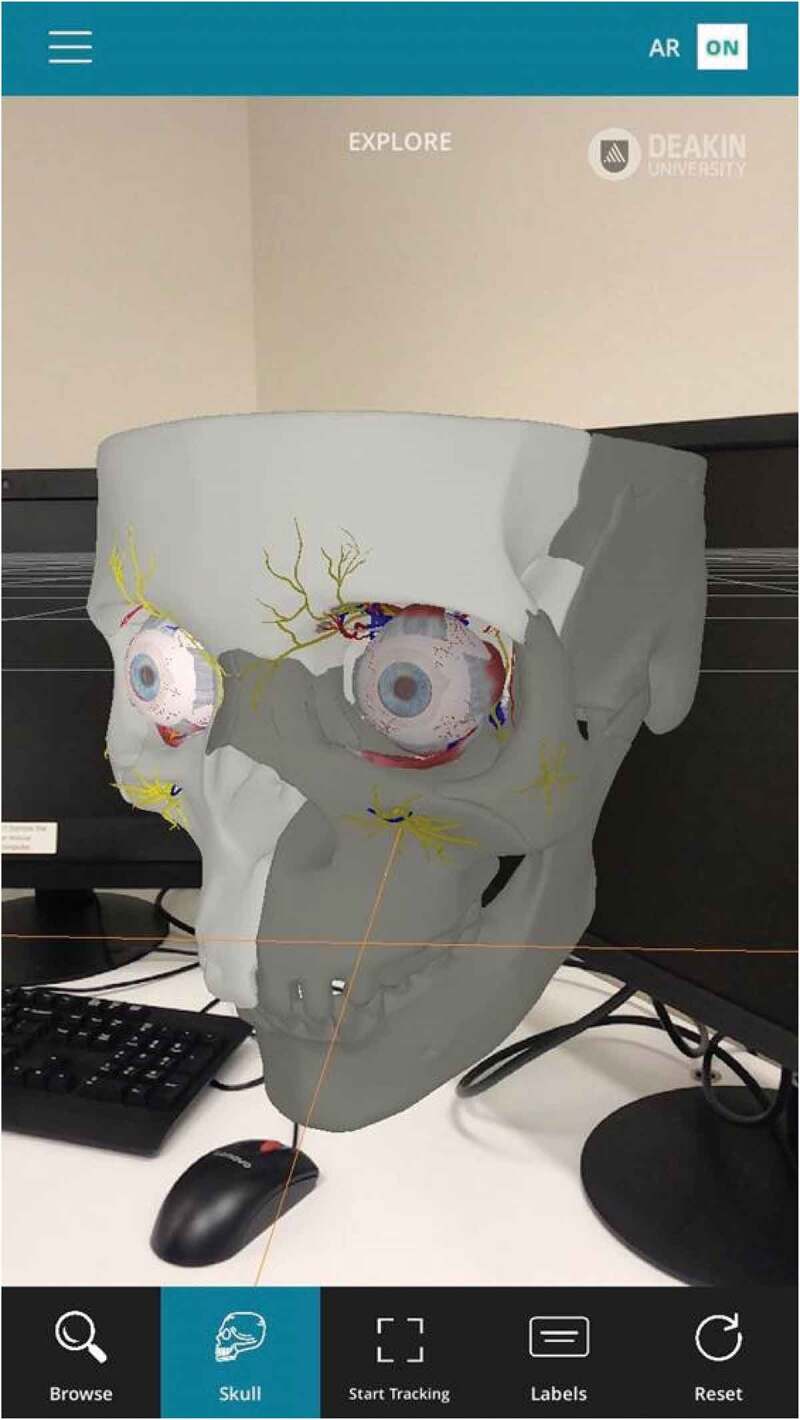
The OculAR SIM AR program to aid optometry students (available on multiple devices, such as tablets and smartphones, subject to licencing conditions). Image courtesy of Apperition (www.appearition.com/deakin-university/), and Peter Bright,School of Medicine, Deakin University

In addition to pure anatomy, understanding how anatomy relates to function is a particularly important aspect of medical education. This is aided by the ease at which different anatomical structures can be added and removed from the digital subject, such as muscles or underlying skeletal structure including muscle attachment sites. Furthermore, many AR (and VR) anatomy programs include functional features where specific muscles can be flexed, in order to observe the resulting movement that they control [25]. This is especially beneficial for understanding complex systems involving multiple muscle groups such as eye movement, which can be accessed easily at the student desk or at home (Figure 4). Another advantage, is that human cadavers and physical models can only logistically represent a limited number of diseased pathologies, and the true range of individual variation is often poorly encapsulated within any given medical school. In contrast, multiple pathologies and subtle anatomical variations can be easily added to virtual representations [26].

### AR enhancement of practical skills

It is an expectation that medicine graduates have inculcated not only extensive and in-depth knowledge about the human body, diseases and associated procedures, but also communication skills, physical examination skills, practical skills and clinical skills. While the current medical pedagogy and curriculum are undoubtedly delivering competency-based medical and healthcare professionals, there is still room for improvement in terms of their practical and surgical skills training; and face-face patient care and treatment is often limited or lacking. Clinical placements have significantly improved the students’ practical skills, but a lack of extensive hands-on training can often hamper their ability to master these procedures [27]. Physical models often help fill this gap, and these are increasingly becoming highly sophisticated and effective. For example, a study conducted in the Medical School of the University of South Carolina used a Cardiopulmonary Patient Simulator called Harvey (i.e., a highly sophisticated manikin), and found that students who were trained for cardiac examination skills using the simulator performed better (on examinations including the USMLE and MCAT) than those trained on standardized manikins or patient models [28]. However, models such as these can be expensive, and the implementation of new resources to improve practical skills is warranted [29].

AR is beginning to make an important contribution to this overall goal, and several cohort studies that confirmed that teaching practical procedures such as surgery using AR-based tools contributes to improvements and practical competence in medical teaching and training. These programs can also often aid the performance of the actual surgical procedure itself, and can overlay patient-specific anatomical information obtained from imaging scans, such as in spinal surgeries [30,31]. Transrectal prostate biopsy practice and training can also be aided by this technology, to help control robot-assisted apparatus [32]. Multiple AR-based programs are already used for kidney surgery and training (and several more are likely to be soon adopted), including those to aid patient education [33,34]. Wolf et al describe the recent development of an AR program using Microsoft HoloLens 2, for training the surgical procedure of extracorporeal membrane oxygenation cannulation. Compared to conventional training, medical students that received the AR version exhibited a higher level of learning and made fewer procedural errors [35]. Another platform has been developed by Nagayo and colleagues for open surgery training. The movement of surgical instruments and patient anatomy during actual procedures was first captured, and then reconstructed within an AR program. Trainees are able to manipulate their view to obtain optimal visual angles, and can pause/rewind the procedure in order to focus on particular stages. Students can also engage in self-practice by manipulating surgical instruments [36].

In addition to AR, VR-based programs are also prominently used to improve practical skills and the performance of surgical techniques. For example, Stanford University introduced the Neurosurgical Simulation and Virtual Reality Center in 2016, which provides medical graduates to explore the structure of the brain and train them to operate [37]. The system has been designed from MRI and CT scans of real patients, offering trainees with an opportunity they would only otherwise get while in the surgery room. They are also able to investigate and operate on a multitude of neurological cases and on virtual cadavers, providing them a real-time experience of the surgical room and how to work under stress and pressure, while being efficient and skilled. Psychomotor skills of surgeons are considered key during laparoscopic procedures, and several programs have been available for a decade or more. For example, a study conducted on surgeons to evaluate the benefits of the Minimally Invasive Surgical Trainer VirtualReality (MISTVR) tool indicates that it polishes their surgical skills [38]. MISTVR offers the added benefits of providing valuable feedback to the trainee students, including parameters such as their handling of the surgical equipment, the duration of the procedure, and possible errors that could have been avoided during the virtual surgery. Several simulation-based platforms for teaching colonoscopy to healthcare professional are also available, such as GI Mentor™ and EndoVR™ (CAE Healthcare, the old AccuTouch®, Immersion) [39]. Hysteroscopy has also been taught for many years using a VR- platform called EssureSim, which has been found to contribute to improved precision amongst the trainees [40]. More recently, VR training was found to be more effective than the standard guide passive learning tool for teaching a complicated tibial shaft fracture surgical technique [41].

### AR enhancement of social skills

Medical training involves extensive learning about social interactions and human behaviour, as future practitioners are expected to perform their duties across a vast spectrum of health care settings. AR provides a unique opportunity to prepare the trainees for complex social situations in a controlled and managed environment. Moreover, AR supports development of inter-professional competencies that are critical for healthcare professionals. Although commonly AR medical training has been viewed as mainly a way to increase knowledge and practical skills, it also provides valuable scenarios to support work-related social skills [42].

Several studies that evaluated the impact of AR in medical training on student experience and learning outcomes assessed inter-professional social competencies, focusing mainly on communication and teamwork. For example, one randomised study of 34 medical residents showed that training in a simulated setting to use endoscopy equipped trainees with better communication skills compared to students who underwent self-regulated learning [43]. Another study investigated endovascular and human factor skills by simulating a crisis scenario that required endovascular ruptured aortic aneurysm repair. The simulation was evaluated by 22 participants with maximum scores for enhancing teamwork and patient safety, with a close second for enhancing team’s communication skills [44].

Some medical students evaluated non-technical skills (stress management such as music in an operating theatre) added to AR training as somewhat destructive and adding to their perceived difficulties in mastering technical skills [45]. However, when a similar stress management situation (telephone calls during a procedure) was tested amongst 19 junior surgeons, the training value of such stimulation was given a mean score of 4.7 out of 5.0. Furthermore, the study results showed that destructive and critical scenarios hinder the objective performance of the surgeons, suggesting these as a valuable addition to training [46].

Another interesting study that showed the value of AR in critical medical training was conducted to prepare operating room clinicians (surgeons, anesthesiologists and nurses) to an event of an operating room fire. Forty-nine participants with a range of clinical experience completed simulation with over two thirds (67%) indicating the preference for AR-based training compared to textbook method [47].

Overall, despite the great potential of AR-based programs to deliver complex and highly precisive training that focusses on social skills building in a vast range of situations, the current evaluation of these in the literature is lacking. A recent systematic review concluded that although simulation-based training in health education is gaining momentum, limited systematic research has been conducted to measure the impact of these on students’ learning outcomes. The majority of the undertaken research to date assessed feasibility and face validity [42]. This outcome shows that although some limited studies indicated great usability of AR training to improve a range of social skills, further comparative investigations are required to evaluate how these could be integrated into the world of virtual medical training.

## Discussion

### Challenges and future directions

Since the first use of AR platforms in orthopaedic diseases, the adoption of this technology in medical education has progressed significantly. One of the major challenges faced by the higher education sector is the cost of designing these interactive platforms [48]. This coupled to the lack of availability of resources to meet the needs of growing student numbers impedes their utilization in medical education. Making this digital technology equitable and accessible to all students is the biggest hurdle faced by educators. Another criticism in the use of AR in teaching is the limited hardware that is needed, in addition to the growing problem of social isolation associated with digital learning [49,50]. Nevertheless, these new digital platforms have enabled educators to push the boundaries of traditional pedagogies to create a student-centric, engaging and enriching the learning experience for the students.

With the advancements in the field, a major next step has been postulated to be the adaptation of AR textbooks in medical education, which is an idea that was proposed almost a decade ago [51]. In this proposal, Yuen (2011) eloquently described how AR books would allow students to transport themselves into a scenario/situation and learn by immersing themselves in the experience. While the subsequent years have seen these books being slowly prescribed in the curriculum at the school level, their incorporation in teaching healthcare and medicine-based courses in higher education is still in its infancy [52,53]. However, continued advancements in this direction could be extremely useful when students are learning about topics including human anatomy and physiology. The ability to read and visualize content such as brain function and nerve impulses (to quote one example) will assist with retention and deeper understanding of the physiology of the human body. When considering the ability of AR designs to allow multiple users to interact in the same platform, this will help tackle the issue of isolation that these AR platforms may create for learners. It is believed that these AR-interfaced books can offer a respite to students from stagnant and dull text-only based learning material that traditional textbooks offer, making them an exciting tool for both students and educators.

**Figure 5.**
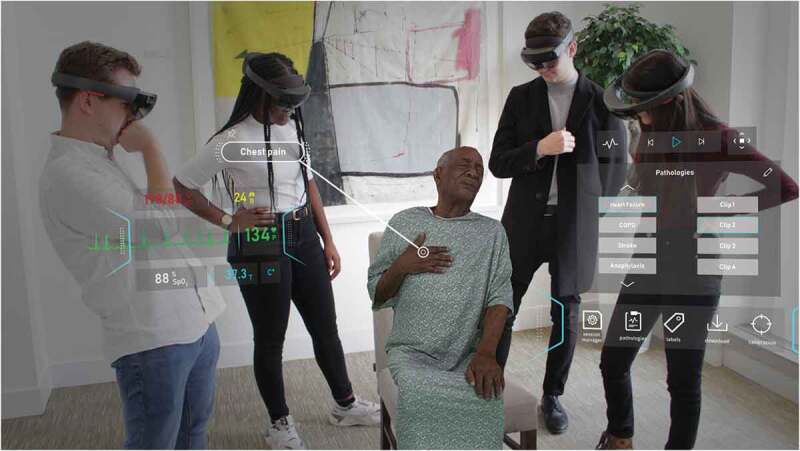
In the HoloPatient system, volumetric 3D video capture of a standardized patient sitting in a chair being assessed by a group of medical students. Students can view the patient and interact with the test results panel and real time vital signs through the use of the Microsoft HoloLens 2. Here the patient describes chest pain associated with myocardial infarction. Published with permission from GIGXR (www.gigxr.com/applications/holopatient)

Mobile learning (m-learning) and wearable technology is a relatively new digital learning platform, that has enormous potential. AR-based learning software could be provided on students’ personal devices such as mobiles, iPads and tablet computers, or on wearable items such as smart watches, which would make the adaptation of this technology in medical education far more acceptable and cheaper [54,55]. One example of this concept is the use of Google Glass [56] at the University of California, Irvine School of Medicine, in their anatomy courses and hospital rotations [57]. Google Glass offers the ease and flexibility of accessing course content and patient-related information in a hands-free format, at the same time allowing users to communicate via voice command. Another wearable technology that can be potentially used in teaching is the use of monitors that can record the health of patients, which is communicated to the smart device of the students, which allows students to detect a disease. For example, The University of Michigan is developing a vapour sensor that can help monitor the health of patients with diabetes and lung disease [58]. The use of virtual patients and case scenarios during problem-based learning sessions is another approach that may be highly beneficial in medical teaching (Figure 5).

Other important potential future uses for AR-based medical education include teaching programs for individuals with reading disabilities (a barrier to traditional textbook-based learning), and in remote learning contexts to transport the user into a virtual space anywhere an internet connection can be made. As AR technologies progress, making these technologies affordable will be a key focus. Collaborations between companies, universities and increased funding for this sector will pave way for newer AR/VR platforms in medical teaching. A classic example is the Medical Virtuality Lab designed by University of Southern California, Institute for Creative Technologies [59]. The primary aim of this institute is to bring individuals and experts from the film and game industry together with computer and social scientists to create and design the platforms for use in healthcare education and training.

The field of AR offers opportunities for educators in the field of medical education to create a rich and engaging curriculum, offering students the opportunity to not only learn but experience the learning content/material as well. The disruption to traditional classroom teaching due to COVID-19 has led to a rapid adaptation of digital teaching tools globally, highlighting the importance of digital technologies, including AR to ensure student learning is not hampered. Optimal utilization and continued usage of digital learning tools has the potential to reform the medical education sector.
